# Functional capacity and actual daily activity do not contribute to patient satisfaction after total knee arthroplasty

**DOI:** 10.1186/1471-2474-11-121

**Published:** 2010-06-16

**Authors:** Maaike M Vissers, Ingrid B de Groot, Max Reijman, Johannes B Bussmann, Henk J Stam, Jan AN Verhaar

**Affiliations:** 1 Department of Orthopaedics, Erasmus University Medical Centre, Rotterdam, the Netherlands; 2 Department of Rehabilitation Medicine, Erasmus University Medical Centre, Rotterdam, the Netherlands

## Abstract

**Background:**

After total knee arthroplasty (TKA) only 75-89% of patients are satisfied. Because patient satisfaction is a prime goal of all orthopaedic procedures, optimization of patient satisfaction is of major importance. Factors related to patient satisfaction after TKA have been explored, but no studies have included two potentially relevant factors, i.e. the functional capacity of daily activities and actual daily activity. This present prospective study examines whether functional capacity and actual daily activity (in addition to an extensive set of potential factors) contribute to patient satisfaction six months after TKA.

**Methods:**

A total of 44 patients were extensively examined preoperatively and six months post surgery. Functional capacity was measured with three capacity tests, focusing on walking, stair climbing, and chair rising. Actual daily activity was measured in the patient's home situation by means of a 48-hour measurement with an Activity Monitor. To establish which factors were related to patient satisfaction six months post surgery, logistic regression analyses were used to calculate odds ratios.

**Results:**

Preoperative and postoperative functional capacity and actual daily activity had no relation with patient satisfaction. Preoperatively, only self-reported mental functioning was positively related to patient satisfaction. Postoperatively, based on multivariate analysis, only fulfilled expectations regarding pain and experienced pain six months post surgery were related to patient satisfaction.

**Conclusions:**

Functional capacity and actual daily activity do not contribute to patient satisfaction after TKA. Patients with a better preoperative self-reported mental functioning, and patients who experienced less pain and had fulfilled expectations regarding pain postoperatively, were more often satisfied.

## Background

Total knee arthroplasty (TKA) is a relatively safe and cost-effective surgical intervention for patients with end-stage osteoarthritis (OA) [1]. After surgery most patients improve with regard to pain and stiffness, self-reported physical functioning and quality of life [2-4]. The greatest improvement is reported within the first six months after surgery [5,6].

Compared to hip arthroplasty, patients undergoing knee arthroplasty show less improvement on the above-mentioned outcome measures and are generally less satisfied [2,3,7]. After TKA only 75-89% of patients are satisfied with their outcome [8-13]. Furthermore, patient satisfaction is one of the ultimate goals of all orthopaedic procedures and, therefore, optimization is of major importance. Knowledge of which factors contribute to patient satisfaction can be incorporated in the care for TKA patients; preoperative and postoperative factors can be used for this purpose. Satisfaction is reported to be associated with self-reported quality of life variables, preoperative mental functioning, expectations and fulfilment of expectations, postoperative pain, and joint stiffness [8,9,13-15]. However, none of these studies included two potentially relevant and modifiable aspects of functioning, i.e. the functional capacity of daily activity and actual daily activity. Studies in other patient groups have shown that these latter factors can be related to quality of life and mental health [16,17]. Therefore, we hypothesise that TKA patients with a higher functional capacity and actual daily activity level will more often be satisfied with the post-TKA results.

This prospective study examines whether functional capacity and actual daily activity, in addition to an extensive set of potential factors, are related to patient satisfaction six months post TKA. Actual daily activity was measured in the patient's home situation by means of a 48-hour measurement with an Activity Monitor (AM) [18]. The study aimed to determine pre- and postoperative factors related to patient satisfaction six months after TKA, focusing on functional capacity and actual daily activity.

## Methods

For the present study, data from a prospective follow-up study (which determined recovery of physical functioning after total knee/hip arthroplasty) were used [19]. In an earlier study by de Groot et al., the pre- and postoperative results focused on recovery of physical functioning after TKA, whereas the present study examines pre- and postoperative results in relation to patient satisfaction six months after TKA. The dependent outcome measure was patient satisfaction six months postoperatively. The following potential factors related to patient satisfaction were measured (on average) six weeks before surgery: functional capacity of daily activity, actual daily activity, gender, age, body mass index (BMI), expectations, joint function (pain, stiffness and strength), self-reported physical functioning, and self-reported mental functioning. The time point of six weeks pre-surgery was used because all patients visit our outpatient clinic about six weeks prior to surgery for preoperative screening. Measurements for the present study were combined with this preoperative screening so that patients visited the outpatient clinic only one time. Six months post surgery the following potential factors related to patient satisfaction were measured: functional capacity, actual daily activity, fulfilled expectations, joint function, self-reported physical functioning and self-reported mental functioning.

All preoperative and postoperative measurements were done by one researcher (IBG) using standardized data collection forms.

### Patient selection

Patients with end-stage OA of the knee who were scheduled for TKA at the Erasmus MC between April 2004 and May 2006 were eligible. Exclusion criteria for the study were: age >80 years, wheelchair-bound, not living independently, the presence of disorders other than OA that could affect the level of actual physical activity, living more than 1.5 h travelling distance from the medical centre, insufficient command of the Dutch language (spoken or written), the presence of OA in the contralateral knee requiring surgery within six months, not willing to sign an informed consent, and not known whether the patient would be available for follow-up measurements. The Medical Ethics Committee of the Erasmus MC approved the study and all patients signed an informed consent form. Patients underwent all measurements (on average) six weeks preoperatively and six months postoperatively.

### Patient satisfaction

Six months after TKA, patient satisfaction with the results of the surgery was assessed: "How satisfied are you with the results of the surgery?" Responses were graded on a 5-point Likert scale: 1 = very satisfied, 2 = moderately satisfied, 3 = neutral, 4 = moderately dissatisfied, and 5 = very dissatisfied. The responses were dichotomised into very satisfied and less satisfied (=moderately satisfied, neutral, moderately dissatisfied, and very dissatisfied) [20].

### Potential factors related to patient satisfaction

#### a) Functional capacity of daily activity

The functional capacity of patients was measured six weeks preoperatively and six months postoperatively using three tests focusing on walking, stair climbing, and sit-to-stand. The Six-Minutes Walk Test (6MWT) was performed to quantify walking ability [21,22]. It is a valid clinical tool that involves recording the maximum distance participants can cover while walking indoors for six minutes. It has good test-retest reliability and has been used to measure the effectiveness of interventions in populations with knee OA [21,22]. Patients were allowed to use walking aids. Preoperatively 26.2% (n = 11) of the patients used walking aids during the 6MWT compared to 9.5% (n = 4) postoperatively.

To assess stair climbing capacity, the time needed for a patient to ascend five steps, turn around, and descend was measured [23]; patients were allowed to use the hand rails.

Chair rising capacity was assessed by measuring how long a patient needed to perform five sit-to-stand movements. Patients were asked to perform this task as quickly as possible [24]; patients were allowed to use their arms while performing the sit-to-stand movements.

#### b) Actual daily activity

Actual daily activity was measured in the home situation of the patient six weeks preoperatively and six months postoperatively. Actual daily activity was measured objectively using an AM [18]. The rationale for the AM sensor configuration, the subsequent steps of the signal analysis, and the method of activity detection have been described in detail previously [18]. In summary, the AM is based on long-term ambulatory (home-based) measurement of signals from body-fixed acceleration sensors (Temec Instruments, Kerkrade, The Netherlands) attached to both upper legs and the sternum. All sensors were connected to a recorder based on Vitaport technology (Temec Instruments, Kerkrade, The Netherlands). The acceleration signals were digitally stored on a flash card. After measurement, all signals were downloaded to a personal computer for further analysis. For each second, a body posture (sitting, standing, lying) or body motion (walking, cycling or other movements) was automatically detected from the acceleration signals. Three aspects of these postures and motions can be distinguished, i.e. quantity, quality and physical strain or motility.

The AM has been extensively validated [18,25-28]. The AM data were collected for 48 h and then averaged. Two AM outcome measures were included: the percentage of time a patient was active per 24 h (walking, cycling, climbing stairs and general movement) and the percentage of time being upright per 24 h (standing and walking).

#### c) Patient-related factors

Preoperatively, gender, age and BMI were determined.

#### d) Expectations and fulfilled expectations

Six weeks preoperatively, the expectations of patients were measured. Assessment of expectations focused on a) pain after surgery b) limitations of activities of daily living after surgery and c) the overall success of the operation. Questions regarding patient expectations of pain and limitations were graded on a 4-point Likert scale (1 = not at all painful/limited, 2 = slightly painful/limited, 3 = moderately painful/limited, and 4 = very painful/limited). The responses were dichotomised: the highest response level was classified as 'high expectations' and the remainder was classified as 'low expectations'. Expectations regarding overall success were recorded on a Visual Analogue Scale (VAS) ranging from 0 -10 (0 indicating no success, and 10 indicating optimal success). These responses were also dichotomised by a VAS score > 9 of success as having 'high expectations' and the remaining scores as 'low expectations'. A similar dichotomisation was applied by Mahomed et al. [29].

Six months post surgery, patients were asked whether their expectations regarding pain, limitations of activities of daily living, and the overall success of the surgery had been fulfilled. Responses of expectations were graded on a 4-point Likert scale (1 = totally fulfilled, 2 = considerably fulfilled, 3 = slightly fulfilled, and 4 = not at all fulfilled). The highest response was classified as 'fulfilled expectations' and the remaining responses were classified as 'unfulfilled expectations' [29].

#### e) Joint function

Joint function was measured six weeks preoperatively and six months postoperatively. The strength of the knee extensors on the affected side was measured with a Microfet hand-held dynamometer [30]. The experienced stiffness was measured using the stiffness subscale of the Western Ontario and McMaster Universities Osteoarthritis Index (WOMAC). Experienced pain was measured using the pain subscale of the WOMAC (0 indicating the worst experienced stiffness/pain, and 100 indicating no experienced stiffness/pain) [31,32].

#### f) Self-reported physical functioning

Self-reported physical functioning was measured six weeks preoperatively and six months postoperatively. The self-reported physical functioning was assessed using the Physical Functioning subscales of the 36-Item Short Form Health Survey (SF-36) [33] and the WOMAC [31,32]. Both subscales range from 0-100, with higher scores indicating better functioning [31,32].

#### g) Self-reported mental functioning

Self-reported mental functioning was measured six weeks preoperatively and six months postoperatively. Self-reported mental functioning was measured using the mental health subscale of the SF-36 [33] and the anxiety and depression subscales of the Hospital Anxiety and Depression Scale (HADS). The HADS consists of 14 items each rated from 0-3 according to severity of difficulty experienced. It is divided into an Anxiety subscale and a Depression subscale. Each subscale score can range from 0-21. The optimal cut-off score for presence of both anxiety and depression disorders is ≥8 [34].

### Statistical analysis

Statistical analysis was performed with SPSS 15.0 (SPSS Inc., Chicago, USA). First, it was established whether the variables had a normal distribution using the normality Shapiro-Wilk test. Based on these analyses, the results are presented as means and standard deviations (SD) or, if not normally distributed, as median (with minimum and maximum).

To establish which factors were related to patient satisfaction six months after TKA, logistic regression analyses were used to calculate odds ratios (OR). Satisfaction six months after TKA was used as dependent variable.

For the univariate logistic regression analysis the six months postoperative factors were adjusted for the preoperative scores using analysis of covariance. Because of the high number of significantly related postoperative factors, multivariate regression analysis was performed to examine which postoperative factors had an independent relationship with patient satisfaction after TKA. We performed multivariate, stepwise (forward) logistic regression. The quality of the multivariate regression models was based on the Akaike Information Criteria (AIC), a measure of the goodness of fit of an estimated model. The AIC aims to find a balance between the conflicting demands of accuracy (fit) and simplicity (small number of variables). Models with smaller AIC are preferred; a difference of two points on the AIC between models should be treated as significantly different models [35]. In all analyses, a two-sided p-value of ≤ 0.05 was considered significant.

## Results

Between April 2004 and May 2006, 45 patients complied with the inclusion criteria and were willing to participate in the study. For one patient only preoperative data are available so, finally, the results of 44 patients were evaluated (Table 1).

**Table 1:** Characteristics of the study population

	Total Population
	n = 44
**Gender, % women (n)**	54.5 (24)
**Age at baseline, years**	63.5 (42.0; 78.0)
**Body mass index at baseline, kg/m^2^**	30.8 (24.2; 44.9)
**Satisfaction regarding overall results**	
- Very satisfied, % (n)	50.0 (22)
- Less satisfied:	
moderately satisfied, % (n)	22.7 (10)
neutral, % (n)	11.4 (5)
moderately dissatisfied, % (n)	9.1 (4)
very dissatisfied, % (n)	6.8 (3)

Values are presented as median (minimum; maximum), unless otherwise indicated

All patients received a Genesis II (Smith and Nephew, Memphis, USA) TKA. Because the Erasmus MC is a university hospital, during the study period the procedures were performed by six different orthopaedic surgeons with different medical assistants. Post surgery, the mean number of days in hospital was 7 (minimum 3, maximum 15) days and all patients received a standard physical therapy and pain management protocol. Three patients had a postoperative complication (neuropraxia, haematoma in the left rectus area, and lung emboli).

In this patient group 50% was very satisfied, 22.7% moderately satisfied, 11.4% neutral, 9.1% moderately dissatisfied and 6.8% very dissatisfied (Table 1). Preoperatively, functional capacity and actual daily activity were not related to patient satisfaction whereas a better self-reported mental functioning was (Table 2). Thus, patients with a better self-reported mental functioning before surgery were more often satisfied post surgery.

**Table 2:** Preoperative factors associated with satisfaction six months post surgery

Determinants	Very satisfied	Less satisfied*	OR (95% CI)	p-value
	n = 22 (50%)	n = 22 (50%)		
***Physical capacity***				
6-MWT (m)	295 (59; 499)	278 (48; 523)	0.999 (0.994; 1.005)	0.844
Stair climbing (s)	11.4 (6; 28)	11.4 (5; 29)	1.000 (0.906; 1.103)	1.000
Rising from chair (s)	18.9 (9; 36)	16.7 (10; 34)	1.047 (0.956; 1.147)	0.317
***Actual daily activity***				
Movement-related activity, %	7.6 (3.8; 17.5)	7.3 (2.7; 17.3)	1.008 (0.861; 1.180)	0.921
of 24 h				
Upright, % of 24 h	17.0 (7.3; 37.2)	21.3 (6.2; 32.7)	0.984 (0.912; 1.062)	0.675

***Patient characteristics***				
Gender, % women (n)	63.6 (14)	45.5 (10)	2.100 (0.628; 7.027)	0.229
Age at baseline, years	57.0 (42; 78)	65.5 (48; 77)	0.957 (0.898; 1.019)	0.169
BMI at baseline, kg/m^2^	30.8 (24; 41)	30.4 (24; 45)	0.962 (0.857; 1.079)	0.567
***Expectations***				
Pain, high expectations, %	9.1 (2)	10.5 (2)	1.176 (0.149; 9.266)	0.877
(number)				
Limitations, high expectations,	27.3 (6)	18.2 (4)	0.593 (0.141; 2.484)	0.593
% (number)				
Overall success, high	45.5 (10)	45.5 (10)	1.000 (0.305; 3.277)	1.000
expectations, % (number)				
***Joint function and structure***				
Strength quadriceps, Newton	145.5 (17; 273)	175 (53; 299)	0.993 (0.983; 1.002)	0.134
Stiffness (WOMAC stiffness)	30 (0; 80)	30 (0; 80)	0.999 (0.968; 1.030)	0.936
Pain (WOMAC pain)	36 (8; 60)	32 (4; 72)	1.009 (0.966; 1.054)	0.680
***Self-reported physical***				
***functioning***				
WOMAC Physical functioning	35.3 (9.4; 62.4)	34.1 (9.4; 63.5)	1.009 (0.964; 1.056)	0.693
SF-36 Physical functioning	32.5 (0; 65)	32.5 (5; 80)	0.997 (0.968; 1.027)	0.827
***Self-reported mental***				
***functioning***				
SF-36 Mental Health	**80 (40; 100)**	**60 (32; 96)**	**1.045 (1.007; 1.085)**	**0.019**
HADS anxiety, % case (n)	**18.2 (4)**	**50 (11)**	**0.222 (0.057; 0.873)**	**0.031**
HADS depression, % case (n)	**13.6 (3)**	**45.5 (10)**	**0.189 (0.043; 0.831)**	**0.027**

Values are presented as median (minimum; maximum), unless otherwise indicated

*Less satisfied = patients who were moderately satisfied, neutral, moderately dissatisfied or very dissatisfied.

OR; Odds Ratio, CI; Confidence Interval, 6-MWT; six-minutes walk test

Significant results are highlighted in bold.

Postoperatively, most factors had a univariate relationship with patient satisfaction. However, actual daily activity showed no association with patient satisfaction, and only the walking test was related to patient satisfaction (Table 3).

**Table 3:** Postoperative factors associated with satisfaction six months post surgery

Determinants	Satisfied	Less satisfied*	OR (95% CI)	p-value
	n = 22 (50%)	n = 22 (50%)		
***Physical capacity***				
6-MWT (m)	**428 (152; 580)**	**368 (132; 485)**	**1.008 (1.001; 1.02)**	**0.034**
Stair climbing (s)	7.3 (5; 18)	9.1 (6.4; 22.8)	0.825 (0.67; 1.02)	0.073
Rising from chair (s)	15 (9.7; 20.8)	16.9 (10; 32.5)	0.872 (0.735; 1.033)	0.113
***Actual daily activity***				
Movement-related activity, %	8.1 (3.2; 17.0)	9.8 (2.8; 18.8)	0.978 (0.839; 1.139)	0.773
of 24 h				
Upright, % of 24 h	20.1 (13.7;39.9)	19.2 (7.2; 30.2)	1.039 (0.936; 1.153)	0.471

***Fulfilled expectations***				
Pain, totally, % (number)	**100 (22)**	**27.3 (6)**	-**	-**
Limitations, totally, %	**68.2 (15)**	**13.6 (3)**	**13.6 (2.99; 61.59)**	**0.001**
(number)				
Overall success, totally, %	**90.9 (20)**	**22.7 (5)**	**34.0 (5.83; 198.15)**	**< 0.0001**
(number)				
***Joint function and structure***				
Strength quadriceps, Newton	216 (156; 307)	195 (106; 293)	1.010 (0.996; 1.023)	0.150
Stiffness (WOMAC stiffness)	**70 (30; 80)**	**40 (0; 80)**	**1.107 (1.04; 1.18)**	**0.001**
Pain (WOMAC pain)	**80 (4; 80)**	**52 (16; 76)**	**1.101 (1.04; 1.18)**	**0.002**
***Self-reported physical***				
***functioning***				
WOMAC Physical functioning	**74.1 (21.2; 80)**	**45.9 (5.9; 74.1)**	**1.112 (1.04; 1.19)**	**0.001**
SF-36 Physical functioning	**77.5 (20; 100)**	**45.0 (10; 90)**	**1.052 (1.02; 1.09)**	**0.003**
***Self-reported mental***				
***functioning***				
SF-36 Mental Health	**92 (36; 100)**	**56 (20; 96)**	**1.076 (1.03; 1.13)**	**0.001**
HADS anxiety, % case (n)	**13.6 (3)**	**45.5 (10)**	**0.189 (0.04; 0.83)**	**0.027**
HADS depression, % case (n)	**9.1 (2)**	**40.9 (9)**	**0.144 (0.03; 0.78)**	**0.024**

Values are presented as median (minimum; maximum), unless otherwise indicated

*Less satisfied; patients who were somewhat satisfied, neutral, somewhat dissatisfied or very dissatisfied.

**Analyses were not performed with SPSS, because 100% had fulfilled expectations in one group.

OR; Odds Ratio, CI; Confidence Interval, 6-MWT; six-minutes walk test

Significant results are highlighted in bold

The multivariate model that best describes the postoperative factors related to patient satisfaction was the model with fulfilled expectations regarding pain and the experienced pain six months post surgery as independent variable.

## Discussion

It was hypothesised that TKA patients with a higher functional capacity and actual daily activity level would more often be satisfied with the post-TKA results. However, the results indicate that functional capacity and actual daily activity (using the specific outcome measures in the present study) do not contribute to patient satisfaction. It is important to note that improving functional capacity and actual daily activity should not only be considered from a patient satisfaction viewpoint. For example, from the health viewpoint it is important to stimulate patients (also post-TKA patients) to be physically active and improve functional capacity.

In addition to functional capacity and actual daily activity, an extensive set of potential factors related to patient satisfaction six months after TKA were examined. Preoperatively, patients with a better self-reported mental functioning were more often satisfied with the results after TKA. Postoperatively (based on multivariate analysis) patients with less experienced pain and fulfilled expectations regarding pain were more often satisfied. The univariate postoperative results showed that perceived physical functioning was related to patient satisfaction. In contrast, actual daily activity had no univariate relationship with patient satisfaction. However, the multivariate results of our study showed that the univariate results should be interpreted with caution, because of the relationships between the examined univariate variables.

Gandhi et al. also found that a poorer preoperative mental health score predicts less satisfaction with surgery after a total joint arthroplasty; however, they combined the results for total hip and knee arthroplasties [14]. Compared with our univariate results, others have shown that postoperative experienced pain and stiffness, fulfilled expectations, and better postoperative self-reported physical and mental functioning are related to patient satisfaction after TKA [8,9,13,15].

The present study focused on the group of very satisfied patients, which was compared with all the other groups (i.e. moderately satisfied, neutral, moderately dissatisfied, very dissatisfied). Therefore, we dichotomised the patients into 'very satisfied' patients and 'less satisfied' patients. Our relatively low satisfaction rate compared to other studies could be explained by the measurement tool used. In the present study, responses regarding the level of satisfaction were graded on a 5-point Likert scale (very satisfied, moderately satisfied, neutral, moderately dissatisfied, and very dissatisfied) whereas in other studies satisfaction was graded on a 2 or 3-point Likert scale (without the answer option "neutral"); consequently, patients had to choose between 'satisfied' or 'not satisfied'. This could have led to differences in satisfaction rates.

Furthermore, our follow-up period of six months was relatively short compared to other studies [8-13]. It is possible that, had our patients made further recovery after six months, this would have led to higher satisfaction rates. On the other hand, a longer follow-up period could result in lower satisfaction rates because patients may realize that no further improvement will be made after six months and become less satisfied. Therefore, the results of our study do not allow to infer whether or not patient satisfaction would increase or decrease had the follow-up period been extended beyond six months.

Our study is unique regarding the investigation of factors potentially related to patient satisfaction after TKA. All patients were extensively examined preoperatively and six months postoperatively. To our knowledge no other studies have examined the relationship between functional capacity and actual daily activity on the one hand, and patient satisfaction after TKA on the other.

However, some limitations of the study need to be addressed. First: the relatively small study population. Patients with end-stage OA of the knee (scheduled for TKA between April 2004 and May 2006) were eligible for inclusion but, due to the strict exclusion criteria, only a relatively small number could be included. This implies that the multivariate regression analyses are mainly explorative and must be interpreted with caution. Future prospective studies should aim for a larger study population. Second: the relatively short follow-up period implies that it remains unclear whether patient satisfaction would be higher or lower in this group had follow-up lasted longer than six months after TKA. On the other hand, the factors influencing patient satisfaction that emerged from the present study were the same as reported in previous studies [8,9,13]. Third: because of our strict inclusion and exclusion criteria and because all patients were recruited from a university hospital, our sample representativeness might have been reduced. However, because the characteristics of our patients and the preoperative and postoperative functioning scores were comparable with other studies [9,13], our sample is probably representative for the TKA population described by others. Another point is that actual performance of daily activity was collected during a relatively short 48-h period. Although a longer data collection period would have been preferred (and some currently available instruments allow this), compared with our AM system these other instruments provide less detailed information on a subject's activities. In contrast to other instruments, the AM not only provides information on the overall activity level, but at each second evaluates the body postures and motions. Therefore, the AM is a unique instrument to measure actual daily activity. Finally, six different surgeons performed the 44 TKAs and it is unknown whether surgeon-related factors influenced the outcome data. However, all TKA procedures were performed under the supervision of four senior surgeons according to a standard protocol with a similar approach and similar implant (Genesis II). Furthermore, the fact that several surgeons performed the TKAs may have increased the generalisability of the results.

The present study examined which factors contribute to patient satisfaction, with the aim to include them (directly or indirectly) in the care for TKA patients. Future studies should further examine the relationship between outcome measures of functional capacity and actual daily activity on the one hand and patient satisfaction on the other hand.

The results of the present study have some clinical implications. Our results suggest that self-reported preoperative mental functioning is related to patient satisfaction after TKA. The question arises whether: a) poor self-reported mental health is mainly caused by the presence of pain and disability and will improve after TKA when the pain and disability has decreased, or that b) interventions designed to reduce preoperative psychological distress should be established to determine whether they might improve patient satisfaction after TKA. Furthermore, our results suggest that pain experienced six months post surgery and fulfilled expectations regarding pain are related to patient satisfaction, both of which are modifiable. The satisfaction level of patients who experience pain after TKA might be increased by optimising post-surgical pain management. Moreover, patient satisfaction might be increased by optimising patient information regarding pain after TKA. These two factors should be targets for future clinical research and implementation.

## Conclusions

Functional capacity and actual daily activity do not appear to contribute to patient satisfaction after TKA. Patients with a better preoperative self-reported mental functioning, and patients who experienced less pain and had fulfilled expectations regarding postoperative pain, were more often satisfied.

## Competing interests

The authors declare that they have no competing interests.

## Authors' contributions

MMV designed the study, collected and analyzed data, and drafted the manuscript. IBG designed the study, and collected and analyzed data. MR designed the study, analyzed data, and helped draft the manuscript. JBB designed the study, analyzed data, and helped draft the manuscript. HJS designed the study. JANV designed the study. All authors ensured the accuracy of the data and analysis, and have read and approved the final manuscript.

## Pre-publication history

The pre-publication history for this paper can be accessed here:

http://www.biomedcentral.com/1471-2474/11/121/prepub
